# The effect of PAP on UACR and metabolic indexes in patients with MS and OSAHS

**DOI:** 10.1007/s11325-024-03044-x

**Published:** 2024-05-08

**Authors:** Fang-jing Shen, Ren-ke Zhou, Dan-qi Qiu, Li Li

**Affiliations:** 1 Ningbo University Health Science Center, Ningbo, Zhejiang China; 2 Department of Endocrinology, the First Affiliated Hospital of Ningbo University, Ningbo, Zhejiang China

**Keywords:** Positive airway pressure, Metabolic syndrome, Obstructive sleep apnea-hypopnea syndrome, Urinary albumin/creatinine ratio

## Abstract

**Purpose:**

To investigate the effects of positive airway pressure (PAP) device on urinary albumin to creatinine ratio (UACR) and metabolic indexes in patients with metabolic syndrome (MS) and obstructive sleep apnea-hypopnea syndrome (OSAHS).

**Methods:**

This study is a retrospective cohort study. Grouped according to whether to use PAP treatment, there were 25 cases in the PAP group and 44 cases in the no OSAHS treatment group. The PAP group received positive airway pressure device and routine treatment of MS. The no OSAHS treatment group received routine treatment of OSAHS and MS. The treatment period is 3 months.

**Results:**

1. The PAP group demonstrated significant reductions in Body Mass Index (BMI), Waist circumference (WC), Neck circumference (NC), Visceral fat area (VFA), Fasting C peptide (FCP), high-sensitivity C-reactive protein (hs-CRP), and UACR compared to the no OSAHS treatment group, with significant differences (P all <0.05). Among them, the UACR in the PAP group decreased significantly (from 86.05(52.55,131.61)mg/g to 16.76(8.70,25.12)mg/g, P<0.001). 2. Linear regression analysis using the decrease in UACR values as the dependent variable demonstrated a positive linear relationship with the decrease in BMI, VFA, fasting insulin (FINS), and homeostasis model assessment of insulin resistance (HOMA-IR). Furthermore, multiple linear regression analysis indicated that the decrease in VFA (B=0.537 [95% confidence interval, 0.084 to 0.989]; *P* = 0.021) and HOMA-IR (B=1.000 [95% confidence interval, 0.082 to 1.917]; *P* = 0.033) values independently correlated with decrease in UACR values.

**Conclusions:**

PAP treatment can reduce UACR in patients with MS and OSAHS, and has the effect of improving metabolic disorders. The decrease of UACR in patients may be related to the decrease of visceral fat and the improvement of insulin resistance.

## Introduction

Due to unhealthy dietary habits, sedentary lifestyles, and irregular work-rest routines, the prevalence of Metabolic Syndrome (MS) in China has seen a steady increase in recent years. According to data from the Chinese Center for Disease Control and Prevention, by 2010, the prevalence of MS among Chinese adults aged over 18 had soared to 33.9%. Shockingly, an estimated 450 million individuals in China suffer from MS [1]. MS represents a cluster of metabolic disorders characterized by the simultaneous onset of obesity, hyperglycemia, dyslipidemia, and hypertension, significantly impacting overall health. It can increase the risk of type 2 diabetes and major cardiovascular events by 2 times and 5 times respectively, and can increase other chronic diseases [2].

Meanwhile, Obstructive Sleep Apnea-Hypopnea Syndrome (OSAHS) stands out as a prevalent sleep-breathing disorder. During sleep, the upper airway of affected individuals repeatedly collapses, leading to intermittent reductions or cessation of ventilation. This occurrence results in hypoxia, hypercapnia, and sleep arousal. Intermittent hypoxia and sleep rhythm disorders caused by OSAHS can also affect the endocrine system and increase the incidence of type 2 diabetes and MS [3].Alarmingly, China has witnessed a surge in OSAHS cases, with an estimated 1.76 million affected individuals, ranking among the highest globally [4].

Indeed, a robust connection exists between MS and OSAHS, and they mutually influence each other. Studies reveal that approximately 60% of individuals with MS also suffer from OSAHS [5]. Conversely, MS is detected in about 40% of patients diagnosed with OSAHS, with the risk of developing MS being nine times higher in those with OSAHS compared to their non-OSAHS counterparts. Furthermore, the risk of MS escalates with the severity of OSAHS [6, 7]. Both MS and OSAHS are systemic diseases capable of inflicting damage on multiple systems, including the renal system. On one hand, various components of MS such as obesity, hyperglycemia, dyslipidemia, and hypertension, can lead to corresponding renal complications including diabetic nephropathy, hypertensive nephropathy, and obesity-associated glomerulopathy [8]. On the other hand, extensive population studies independently abnormal presence of excess proteins in urine. Simultaneously, individuals with proteinuria exhibit a higher prevalence of sleep apnea [9]. Urinary microalbumin is a sensitive indicator of early renal damage. The gold standard for measuring microalbuminuria is the collection of a 24-hour urine sample, but this process is cumbersome. Additionally, using spot urine collection can be influenced by changes in urine volume and concentration, affecting the measurement of urinary microalbumin. The use of urine albumin-to-creatinine ratio (UACR) can correct for the impact of urine concentration and volume on test results [10]. Research by Gansvoort et al. [11] indicates that there is no significant difference in assessing microalbuminuria between 24-hour urine albumin concentration and UACR. The American Diabetes Association also recommends using UACR for screening and diagnosing microalbuminuria. UACR<30μg/mg is defined as normal, 30≤UACR<300μg/mg as microalbuminuria, and UACR≥300μg/mg as macroalbuminuria. Specifically, Patients with OSAHS often manifest microproteinuria, and their UACR is significantly elevated compared to the general population [12].

Positive Airway Pressure (PAP) stands as the primary treatment for patients with OSAHS. This therapeutic equipment administers a continuous positive pressure airflow, transmitting it into the body to support the patient's airway. By establishing an air scaffold, it effectively prevents the collapse of the airway during the patient's inhalation. The objective is to reduce the Apnea-Hypopnea Index (AHI), ameliorate hypoxia, and alleviate clinical symptoms, such as daytime fatigue and somnolence [13]. Given China’s substantial population, there exists a considerable number of patients grappling with both MS and OSAHS often accompanied by early signs of renal injury. Therefore, investigating the efficacy of PAP in this specific cohort carries immense significance. This study aims to explore the impact of PAP on UACR in patients with MS and OSAHS, delving into its effectiveness in managing MS. The findings are poised to contribute valuable insights and theoretical foundation for the subsequent treatment of individuals contending with both MS and OSAHS.

## Methods

### Study design

**Fig. 1:**
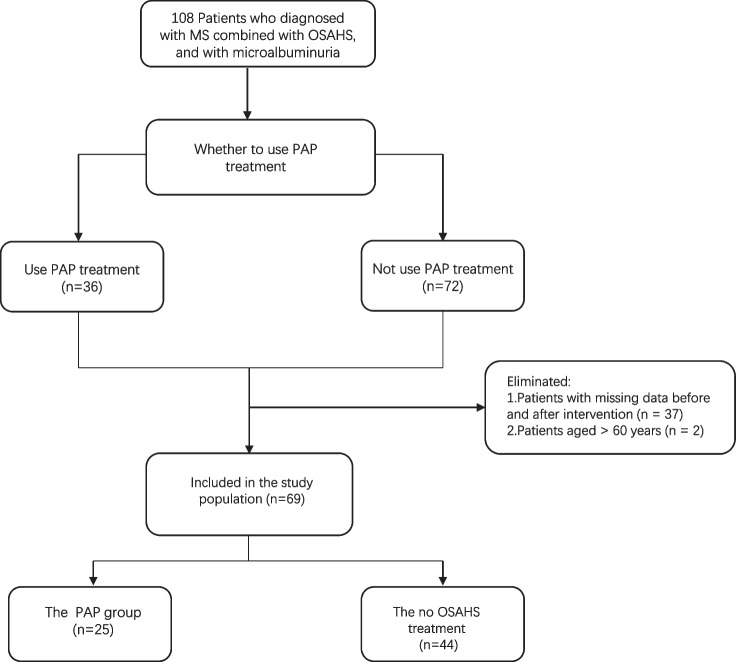
Flow chart of cohort integration

This retrospective cohort study involved 108 MS patients who attended the outpatient clinic of the Department of Endocrinology at the First Affiliated Hospital of Ningbo University. In the data analysis statistics, 11 patients were excluded due to the lack of baseline UACR indicators, 26 patients were excluded due to the lack of UACR indicators after intervention, and 2 patients were excluded due to age over 70 years old. Finally, 69 patients were included in the study (Fig.1). The study period spanned from July 2020 to December 2022. Patients were diagnosed with OSAHS through portable sleep apnea monitoring and exhibited microalbuminuria. All participants were enrolled in the national standardized metabolic management center program. Ethical considerations were addressed, and the study received approval from Ethics Committee of the First Affiliated Hospital of Ningbo University. Informed consent was obtained from all patients, adhering to the principles outlined in the Declaration of Helsinki.

### Participants

The enrolled participants in the study were individuals aged between 18 and 70 years who had not previously undergone noninvasive positive pressure ventilation including PAP. The inclusion criteria for participants selection were based on specific diagnostic criteria for MS and OSAHS. MS diagnosis was determined according to the criteria outlined in the Chinese Guidelines for the Prevention and Control of Type 2 Diabetes Mellitus (2020 edition) [14]. Additionally, OSAHS diagnosis followed the Guidelines for Primary Diagnosis and Treatment of Obstructive Sleep Apnea in Adults(2018) [15]. Specifically, participants needed to exhibit a measured UACR between ≥30 mg/g and <300 mg/g. Exclusion criteria were applied to ensure a homogeneous study population. Individuals with comorbidities such as simple snoring, or diseases that affect sleep quality, such as narcolepsy, restless leg syndrome, post-traumatic stress disorder, and periodic limb movement disorders, or with a history of severe cardiac, hepatic, or renal insufficiency, a history of malignant tumors, and the use of atypical antipsychotics, antiepileptics, chronic steroids, and those experiencing pregnancy or lactation were excluded from the study. Furthermore, any other conditions that were deemed incompatible with study objectives were also considered as exclusion criteria.

### Procedures

The 69 patients were divided into two groups: the PAP group, consisting of 25 patients, and the no OSAHS treatment group, with 44 patients. During the baseline visit, patients with pre-existing microalbuminuria received targeted interventions following clinical guidelines. These interventions included measures such as avoiding a high-protein diet and the addition of angiotensin-converting enzyme inhibitor (ACEI) or angiotensin II receptor antagonist (ARB) drugs. All patients, regardless of group assignment, received conventional treatment for MS. This comprehensive approach involved lifestyle interventions, such as adopting a proper diet, engaging in appropriate physical activity, and weight management. Additionally, various components of MS, such as blood pressure, blood glucose, and dyslipidemia, were controlled suitable medications.

In the PAP group, patients utilized the Philips Respironics DreamStation Auto PAP, a non-invasive positive pressure ventilation device commonly used in hospitals. A sleep apnea therapist guided by sleep monitoring report, fitted a nasal mask based on individual factors such as weight, face shape and nose size. They then adjusted pressure parameters through a trial process to ensure the patients’ adaptation to the ventilator. Patients in this group were also provided with the DreamMapper App on their cell phones, which allowed them to access data related to their PAP therapy, including information on ventilator use time, AHI, and mask leakage. Educational videos on the app facilitated patients understanding of ventilator use and OSAHS treatment. For monitoring and assessment, the physician's end was equipped with EncoreAnywhere, a cloud-based patient data management system. This system enabled physicians to monitor the use of the patient's noninvasive ventilator and assess adherence to home PAP therapy. In contrast, the no OSAHS treatment group received routine OSAHS treatment, which included smoking and alcohol cessation, weight control, and adopting a side-lying sleep position.

Patients underwent a thorough examination both at baseline and three months post-treatment. A comprehensive assessment of anthropometric characteristics was conducted during each visit, encompassing measurements such as height, weight, waist circumference (WC), neck circumference (NC), visceral fat area (VFA), resting blood pressure and body mass index (BMI). Detailed information regarding patients’ medical history, current medications, dietary habits and exercise routines in daily life was meticulously recorded through in-depth interviews. Each visit involved a minimum 8-hour fasting period for the patients. Peripheral venous blood samples were systematically collected on an empty stomach in the morning of the same day. Additionally, midstream urine samples were obtained for an array of crucial test items. These included fasting plasma glucose (FPG), fasting C-peptide (FCP), fasting insulin (FINS), glycated hemoglobin (HbA1c), triglyceride (TG), total cholesterol (TC), high-density lipoprotein cholesterol (HDL-C), low-density lipoprotein cholesterol (LDL-C), serum creatinine (Scr), high-sensitivity C-reactive protein (hs-CRP), urinary microalbumin (U-ALB), and UACR. For non-insulin users, insulin resistance was calculated by the HOMA-IR (Homeostatic Model Assessment for Insulin Resistance) index as FPG (mmol/L) × FINS (mIU/L)/22.5. The association between the risk of OSAHS and MS was explored through the continuous MS severity score. This score, denoted as the Mets Z-score, was computed using equations developed for Korean adults [16].

### Outcomes

The primary efficacy indicators were the changes of UACR and MetS Z-score from baseline to the last measurement during treatment. Secondary indicators recorded from baseline to 3 months of intervention included BMI, WC, NC, VFA, UA, U-ALB, Scr, blood pressure, glucose and lipid metabolism-related indicators.

### Statistical analysis

SPSS25.0 software was used for statistical analysis. All measurement data are first tested for normality. The measurement data of normal distribution were described by mean ± standard deviation (x±SD). Two independent sample t-tests were used for comparison between groups, and paired sample t-tests were used for comparison within groups. The measurement data that did not meet the normal distribution were expressed as quartiles (p25, p75). The non-parametric test was used for comparison between groups, and the rank sum test was used for comparison before and after the group. Spearman correlation analysis was used to analyze the correlation of non-normal distribution data. The influencing factors of UACR decline were analyzed by linear and multiple linear regression analysis. *P*< 0.05 was considered statistically significant.

## Results

In this study, a total of 69 patients were included, comprising 25 patients in the PAP group, consisting of 20 males and 5 females, with an average age of 38.0(32.5,54.0) years. The no OSAHS treatment group comprised 44 cases with 30 males and 14 females, and an average age of 36.0(29.0,50.25) years. Prior to treatment, a statistically significant difference in AHI was observed between the two groups (*P*<0.05), indicating that the AHI in the PAP group was higher than that in the no OSAHS treatment group. However, there were no significant differences in sex, age, basic diseases situation and drug usage between the two groups (*P*>0.05) (Table 1).

**Table 1:** Comparison of baseline characteristics between the two groups

Characteristic	PAP group (*n*=25)	no OSAHS treatment group (*n*=44)	*P* Value
Age(yr)	38.00(32.50,54.00)	36.00(29.00,50.25)	0.476
Sex (males, %)	20(80.00)	30(68.18)	0.291
AHI (times/h)	48.10(26.70,60.10)	20.20(13.73,39.65)	0.002*
Basic disease situation			
Hypertension (%)	19(76.00)	31(70.45)	0.620
Diabetes (%)	18(72.00)	37(84.09)	0.230
lipid abnormality (%)	22(88.00)	39(88.64)	1.000
abdominal obesity (%)	24(96.00)	44(100.00)	0.773
Drug use			
ACEI/ARB (%)	14(56.00)	24(54.55)	0.907
GLP-1RA (%)	14(56.00)	27(61.40)	0.663
SGLT-2 inhibitors (%)	12(48.00)	27(61.40)	0.282

*Definition of abbreviations: AHI* apnea hypopnea index, *ACEI* angiotensin converting enzyme inhibitor, *ARB* angiotensin II receptor antagonist, *GLP-1RA* glucagon-like peptide-1 receptor agonist, *SGLT-2* sodium-glucose cotransporter 2, *BMI* body mass index

The baseline characteristics of the two study groups were similar (*P*>0.05). After a three-month follow-up, patients in both groups exhibited significant reductions in BMI, SBP, DBP, WC, NC, VFA, FPG, HbA1c, HOMA-IR, TG, TC, LDL-C, UA, MetS Z-score, U-ALB, and UACR compared to baseline values. However, in the PAP group, patients also showed decrease in FCP, FINS, and hs-CRP, while the no OSAHSA treatment group exhibited a decrease in HDL-C (*P*_*2*_=0.007) and an increase in Scr (*P*_*2*_<0.001). A comparison between the two groups revealed that the PAP group had more significant reductions in BMI, WC, NC, VFA, FCP, hs-CRP, and UACR compared to the no OSAHS treatment group (*P*_*3*_<0.05). Although PAP patients did not show a pronounced improvement in the severity of MS, the reduction in MetS Z-score was greater than that observed in the no OSAHS treatment group (Table 2). In addition, we collected the average AHI value of PAP group after 3 months and the percentage of days of using time ≥ 4 hours through the EncoreAnywhere system. The data showed that the average AHI of 25 patients decreased significantly to 3.10(1.95,4.80) times/hour after PAP treatment (P<0.001), and their average percentage of using PAP ≥ 4 hours per night was 88.9%.

**Table 2:** Descriptive characteristics at baseline and following the intervention by group

Characteristic	PAP group (*n*=25)		*P*_*1*_ *Value*	no OSAHS treatment group (*n*=44)		*P*_*2*_ value	*P*_*3*_ Value
	Baseline	After		Baseline	After		
BMI (kg/m^2^)	31.30(26.70,41.20)	28.50(25.20,33.80)	<0.001*	32.15(27.93,37.80)	30.30(26.40,35.78)	<0.001*	0.039*
SBP (mmHg)	148.32±20.02	136.52±11.11	0.003*	145.89±17.85	136.55±17.04	0.001*	0.349
DBP (mmHg)	94.72±13.80	85.56±8.90	<0.001*	88.93±12.35	81.43±8.09	<0.001*	0.463
WC (cm)	103.00(94.00,121.50)	101.90±16.56	<0.001*	102.25(93.63,113.75)	101.10±14.44	0.001*	0.021*
NC (cm)	43.00(39.00,45.25)	39.96±4.41	<0.001*	41.00(38.63,43.75)	39.73±4.25	<0.001*	0.019*
VFA (cm^2^)	163.00(121.45,191.60)	115.00(84.50,162.65)	<0.001*	154.00(106.50,190.80)	129.00(94.00,169.00)	<0.001*	0.002*
FPG (mmol/L)	6.74(6.03,9.23)	5.54(5.11,5.87)	<0.001*	7.46(5.97,11.15)	5.44(5.01,6.53)	<0.001*	0.389
HbA1c (%)	6.50(5.95,7.95)	5.50(5.25,5.95)	<0.001*	7.05(5.95,9.78)	5.75(5.20,6.78)	<0.001*	0.965
FCP (nmol/L)	1.32±0.56	0.99(0.72,1.15)	<0.001*	1.42±0.55	1..27(0.94,1.92)	0.966	0.011*
FINS (pmol/L)	128.50(79.05,209.45)	76.66(59.53,117.28)	<0.001*	161.75(112.80,240.60)	131.02(76.42,208.05)	0.183	0.330
HOMA-IR	6.71(3.72,8.84)	2.93(1.93,4.06)	<0.001*	8.91(4.50,13.15)	4.47(2.56,7.42)	0.002*	0.736
TG (mmol/L)	2.51(1.65,3.21)	1.45(1.10,1.95)	<0.001*	1.98(1.60,3.11)	1.46(0.97,2.06)	<0.001*	0.182
TC (mmol/L)	5.74±1.42	4.07(3.61,4.75)	<0.001*	5.55±1.35	4.16(3.58,4.82)	<0.001*	0.618
LDL-C (mmol/L)	3.63±0.96	2.53(1.99,3.00)	<0.001*	3.73±1.00	2.66(2.19,3.26)	<0.001*	0.512
HDL-C (mmol/L)	1.18(1.03,1.29)	1.18(0.96,1.40)	0.788	1.12(0.96,1.28)	1.08(0.89,1.18)	0.007*	0.129
UA (μmol/L)	435.80(349.50,495.25)	358.28±102.95	0.002*	374.85(326.33,469.40	330.65±70.07	0.003*	0.499
hs-CRP (mg/L)	1.15(0.54,2.80)	0.50(0.50,0.83)	0.030*	0.73(0.50,2.24)	0.51(0.50.1.91)	0.509	0.040*
Scr (μmol/L)	67.00(53.00,76.50)	67.00(57.00,83.00)	0.396	60.00(50.50,70.75)	70.00(60.00,80.75)	<0.001*	0.009*
U-ALB (mg/l)	139.20(75.17,192.87)	26.30(14.90,41.30)	<0.001*	90.85(47.93,194.40)	36.50(13.88,74.75)	<0.001*	0.110
UACR (mg/g)	86.05(52.55,131.61)	16.76(8.70,25.12)	<0.001*	69.44(40.01,126.08)	23.03(9.30,55.38)	<0.001*	0.013*
MetS Z-score	2.68±1.33	1.16±1.06	<0.001*	2.37±1.32	1.55±0.94	<0.001*	0.283

*Definition of abbreviations: BMI* body mass index, *SBP* systolic blood pressure, *DBP* diastolic blood pressure, *WC* waist circumference, *NC* neck circumference, *VFA* visceral fat area, *FPG* Fasting plasma glucose, *HbA1c* glycated hemoglobin, *FCP* fasting C peptide, *FINS* fasting insulin, *HOMA-IR* homeostasis model assessment of insulin resistance index, *TG* triglyceride, *TC* total cholesterol, *LDL-C* low density lipoprotein cholesterol, *HDL-C* high-density lipoprotein cholesterol, *UA* uric acid, *hs-CRP* high-sensitivity C-reactive protein, *Scr* serum creatinine, *U-ALB* urinary microalbumin, *UACR* urinary microalbumin to creatinine ratio, *MetS Z-score* continuous variables for MetS severity. *P*_*1*_ value represents the comparison of indicators before and after treatment in the PAP group; *p*_*2*_ value represents the comparison of indicators before and after treatment in the no OSAHS treatment group; *p*_*3*_ value represents the comparison of the difference between the two groups before and after treatment

In order to further analyze the influencing factors of reducing UACR, spearman correlation analysis was performed between the change value of UACR and the use of PAP, age, sex and anthropometric indicators before treatment. It was found that the improvement of UACR was positively correlated with the use of PAP (*P*=0.011), neck circumference (*P*=0.009), TC (*P*=0.032) and LDL-C (*P*=0.013) before treatment (Table 3). The pre-treatment value of an indicator in the group minus the post-treatment value is defined as the difference between the treatment indicators, and the letter d is added to the front to distinguish (e.g., dBMI=pre-treatment BMI-post-treatment BMI). In the combined analysis of the two groups, it showed that the improvement of UACR was also positively correlated with dBMI, dNC, dVFA, dHbA1c, dFINS, dHOMA-IR and dhs-CRP (*P*<0.05).

**Table 3:** Spearman correlation analysis of UACR reduction in two groups

Characteristic	correlation coefficient	*P* Value
Age(yr)	-0.177	0.146
Sex	0.036	0.770
Use PAP	0.303	0.011*
BMI (kg/m^2^)	0.152	0.214
SBP (mmHg)	-0.086	0.480
DBP (mmHg)	0.166	0.173
WC (cm)	0.162	0.183
NC (cm)	0.311	0.009*
VFA (cm^2^)	0.199	0.104
FPG (mmol/L)	0.015	0.905
HbA1c (%)	0.131	0.282
FCP (nmol/L)	0.093	0.448
FINS (pmol/L)	0.121	0.324
HOMA-IR	0.077	0.527
TG (mmol/L)	0.039	0.750
TC (mmol/L)	0.258	0.032*
LDL-C (mmol/L)	0.298	0.013*
HDL-C (mmol/L)	-0.085	0.485
hs-CRP (mg/L)	0.103	0.398
Scr (μmol/L)	-0.002	0.985
MetS Z-score	0.098	0.422

The abbreviations are the same as Table 2

**Fig. 2:**
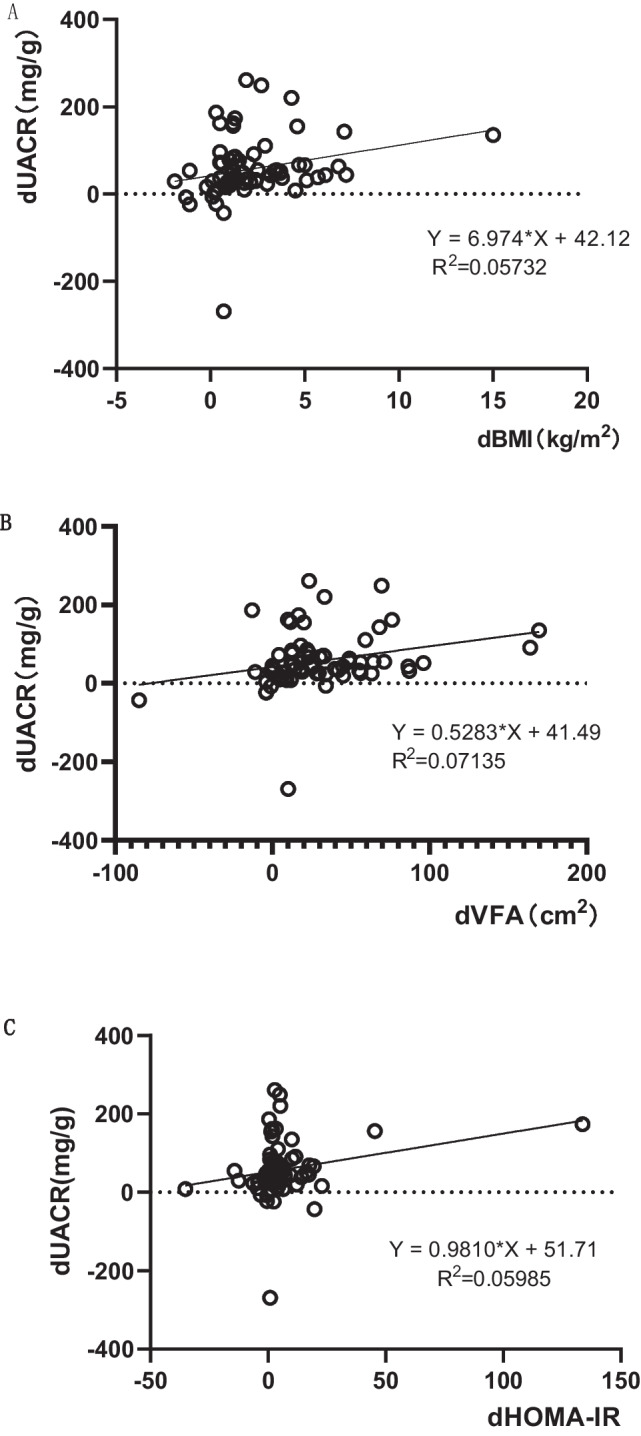
Relationship of dUACR with dBMI, dVFA, dHOMA-IR. (**A**) The linear regression relationship between dBMI and dUACR. (**B**) The linear regression relationship between dBMI and dVFA. (**C**) The linear regression relationship between dBMI and dHOMA-IR

With dUACR as the dependent variable and the indexes related to dUACR as the independent variables, the linear regression analysis was included respectively. Fig. 2 shows that there were positive correlations of dUACR with dBMI (*P*=0.048, *r*=0.239), dVFA (*P*=0.026, *r*=0.267), and dHOMA-IR (*P*=0.043, *r* =0.245)

Furthermore, multiple linear regression analysis (step by step method) was performed with dUACR as the dependent variable and dBMI, dNC, dVFA, dHbA1C, dFINS, dHOMA-IR and dhs-CRP as independent variables. The results showed that there was a positive linear correlation between dVFA (B=0.537), dHOMA-IR (B=1.000). Besides, dVFA and dHOMA-IR were independent factors affecting dUACR (*P*<0.05) (Table 4).

**Table 4:** Multivariate linear regression of the decreased value of UACR

Characteristic	B value (95%CI)	t value	*P* Value
dVFA (cm^2^)	0.537 (0.084~0.989)	2.368	0.021*
dHOMA-IR	1.000 (0.082~1.917)	2.176	0.033*

The abbreviations are the same as Table 2. d means the pre-treatment value of an indicator in the group minus the post-treatment value

## Discussion

The presence of a minimal amount of protein in urine, known as microalbuminuria, signifies an early indication of kidney disease and is often considered a marker of endothelial dysfunction. OSAHS not only contributes to proteinuria, but also correlates with the disease’s severity and the extent of urinary protein excretion. Faulx et al. [17] investigated the urinary protein excretion rates among 496 patients across varying degrees of OSAHS. Their findings revealed a positive correlation between AHI and UACR. Remarkably, this correlation persisted even after excluding subjects with renal insufficiency. Another study compared UACR levels between OSAHS patients and individuals with simple snoring. It was found that OSAHS can induce microalbuminuria, and the severity of this condition correlates with the extent of hypoxemia experienced by the patients [18]. A meta-analysis confirmed an associated between OSAHS and heightened levels of proteinuria alongside reduced eGFR [19]. In the analysis conducted by Liu et al. [12] examining the link between OSAHS and early renal damage, results demonstrated a significant reduction in eGFR among OSAHS patients compared to healthy individuals. This reduction was notably more pronounced in patients with concurrent hypertension and/or diabetes. The above results underscore the detrimental impact of OSHAS on renal function, leading to proteinuria and decreased eGFR. Moreover, individuals affected by both OSAHS and MS exhibit more severe renal impairment compared to those solely diagnosed with OSAHS. Research on the renal damage associated with MS and OSAHS has been primarily limited to exploring either one of the conditions independently. However, considering the interrelated nature of MS and OSAHS, their combined influence likely exacerbates the likelihood of microalbuminuria occurring during the early stage of the disease.

Therefore, this study investigated the influence of PAP treatment on urinary microalbumin levels in MS patients with coexisting OSAHS and microalbuminuria. The primary focus was on the impact of PAP treatment on UACR to mitigate the potential effects of variations in urine volume and concentration. To minimize confounding variables related to baseline medications, a thorough statistical analysis was conducted on drugs known to significantly reduce urinary protein in both patient groups. The investigation revealed no significant difference in the use of ACEI/ARB drugs, GLP-1 receptor agonists, and SGLT-2 inhibitors between the two groups. Results demonstrated a notable discrepancy in UACR and Scr levels before and after treatment(*P*<0.05). Specifically, the PAP group exhibited a more substantial reduction in UACR, whereas the no OSAHS treatment groups experienced a higher Scr level after three months, so it was considered that PAP had a positive effect on diminishing microalbuminuria and enhancing renal function.

Spearman correlation analysis was conducted to examine the relationship between the decrease in UACR, the use of PAP, and various baseline patients’ data. The results revealed a positive correlation between the reduction in UACR and the use of PAP, as well as baseline parameters such as NC, TC, LDL-C, AHI before treatment. Patients with more severe OSAHS, higher blood lipids and greater NC before intervention, especially those using PAP, exhibited more pronounced reduction in UACR. which suggested that these patients can achieve better benefits in reducing urinary protein. The potential causes of the observed decrease in UACR after treatment were further analyzed. The results indicated a positive correlation between the reduction in UACR and decreases in BMI, NC, VFA, HbA1c, FINS, HOMA-IR and hs-CRP. This suggested that weight loss, reduction in NC, lower HbA1c levels, improved HOMA-IR, and a decrease in microinflammatory state may contribute to the decrease in UACR. Further linear regression analysis with UACR decline value as the dependent variable showed that the decrease of BMI, VFA, FINS, HOMA-IR were positively linearly correlated with UACR decline. Multiple linear regression analysis identified HOMA-IR and VFA decline as independent factors influencing the reduction in UACR.

In addition, in the comparison of the difference between the two groups before and after treatment, the PAP group exhibited a more significant decrease in WC, NC, VFA, FCP and hs-CRP compared to the no OSAHS treatment group. Obesity, heightened visceral fat, and insulin resistance are closely related, and these factors have been linked to the onset of proteinuria. Intermittent hypoxemia resulting from OSAHS can exacerbate stress and activate inflammatory pathways, ultimately leading to endothelial dysfunction and proteinuria. In summary, this study suggested that the mechanism by which PAP reduced urinary protein may be associated with mitigating chronic low-grade inflammation induced by hypoxia in the body. This reduction was accompanied by a decrease in body weight, visceral fat, and an improvement in insulin resistance. Notably, the study underscored the potential importance of reducing visceral fat and improving insulin resistance in the overall efficacy of PAP treatment in alleviating proteinuria.

In alignment with the finding of our study, a multicenter investigation led by Zamarron et al. [20] found that a 52-week course of continuous positive airway pressure (CPAP) treatment was linked to a notable reduction in UACR and improvement in insulin resistance among patients. Yasar et al. [21] showed that even a short-term, one-month CPAP treatment could significantly decrease the urinary albumin excretion rate and UACR. Another study by Daskalopoulou et al. [22] observed that CPAP could reduce sympathetic nerve activity and alleviate proteinuria in OSAHS patients, with this effect persisting for a duration of three months. A meta-analysis further supported these findings, indicating a significant reduction in UACR in patients with OSAHS treated with CPAP [23]. In our investigation focusing on microalbuminuria in MS patients with OSAHS, PAP treatment emerged as a significant factor in reducing UACR. Correlation analysis underscored the relationship between the use of PAP and the decrease in UACR. However, multiple linear regression did not show PAP as an independent factor in reducing UACR. This limitation may be attributed to the study’s small sample size, variations in baseline medication regimens, and other confounding factors. It was crucial to note that while PAP was primary intervention for OSAHS, it may not be the exclusive or paramount treatment for reducing proteinuria in clinical practice. Despite these limitations, this study provided a foundation basis for further research in this area.

Prior researches has consistently demonstrated a substantial association between OSAHS and MS, revealing abnormalities in lipid metabolism [24], the induction of refractory hypertension [25], and an elevated risk of type 2 diabetes [26, 27]. Additionally, OSAHS can exacerbated the severity of MS, although greater physical activity may help mitigate this risk [28]. PAP treatment emerges as a promising intervention, enhancing insulin resistance, glucose metabolism and lowering blood pressure by addressing nocturnal intermittent hypoxia, reducing oxidative stress, and modulating the sympathetic nervous system, among other mechanisms [29, 30]. Various Studies indicate that CPAP treatment not only reduces blood pressure but also ameliorates endothelial dysfunction [31] and atherosclerosis [32]. The impact on lipid metabolism, however, varies across studies. Simon et al. [33] reported a significant reduction in TC and LDL-C with CPAP, while another study showed an increase in HDL-C [34]. A meta-analysis also obtained similar results, revealing decreased TC and TG, increased HDL-C, but no significant effect on LDL-C [35]. In the context of our study, post-treatment analysis revealed that levels of FCP, FINS and HOMA-IR in the PAP group were significantly lower than those in the no OSAHS teatment group (*P*< 0.05)., which suggested that the PAP group exhibited superior blood glucose control, with a more pronounced improvement in insulin resistance. Although there was no significant difference in the improvement of blood pressure, blood lipid profiles and MS severity between the two groups, the reduction in these indicators within the PAP group was greater than that observed in the no OSAHS treatment group. This implied that while PAP may not exert a conspicuous effect on improving MS itself, it dose contributed significantly to improve associated metabolism.

### Limitations

The sample size of this study is limited, and the research period is relatively short. There was a statistically significant difference in AHI between the PAP group and the non-PAP group. This will affect the results. Additionally, it is a retrospective cohort study, which may compromise the statistical validity of the research findings. Regarding the examination methods, a majority of patients underwent diagnosis and severity assessment of OSAHS using a portable sleep breathing monitor. However, the results obtained from portable sleep breathing monitoring at home may not be as accurate as those from polysomnography conducted in a hospital setting. Currently, CT or MRI is considered the gold standard for determining visceral fat. Using the bioelectrical impedance method to assess the VFA of patients might introduce some errors. Furthermore, the study utilized HOMA-IR to evaluate the insulin sensitivity of patients; However, employing the glucose clamp test could provide more accurate results. It’s worth noting that the study’s population is mainly from Ningbo City, Zhejiang Province. To enhance the generalizability of the findings, further research should involve a more diverse population and employ a multi-center, prospective approach with a larger sample size.

## Conclusion

PAP treatment can reduce UACR in patients with MS and OSAHS, and has the effect of improving metabolic disorders. The decrease of UACR in patients may be related to the decrease of visceral fat and the improvement of insulin resistance.

## Data Availability

The datasets used and/or analyzed during the current study are available from the corresponding author on reasonable request.
